# The role of indigenous health workers in promoting oral health during pregnancy: a scoping review

**DOI:** 10.1186/s12889-018-5281-4

**Published:** 2018-03-20

**Authors:** Ariana C. Villarosa, Amy R. Villarosa, Yenna Salamonson, Lucie M. Ramjan, Mariana S. Sousa, Ravi Srinivas, Nathan Jones, Ajesh George

**Affiliations:** 1 Centre for Oral Health Outcomes, Research Translation and Evaluation (COHORTE), Ingham Institute for Applied Medical Research, Locked Bag 7103, Liverpool BC, NSW 1871 Australia; 2 School of Nursing & Midwifery, Western Sydney University, Penrith, 2751 Australia; 3 South Western Sydney Local Health District, Liverpool, NSW Australia; 4 Ingham Institute for Applied Medical Research, Liverpool, 1871 Australia; 5 Centre for Applied Nursing Research (CANR), Liverpool, 1871 Australia; 6 Prince of Wales Clinical School, University of New South Wales, Sydney, 2052 Australia; 7 Faculty of Dentistry, University of Sydney, Camperdown, 2050 Australia

**Keywords:** Indigenous, Aboriginal, Health workers, Oral health, Antenatal

## Abstract

**Background:**

Early childhood caries is the most common chronic childhood disease worldwide. Australian Aboriginal and Torres Strait Islander children are twice more likely to develop dental decay, and contributing factors include poor maternal oral health and underutilisation of dental services. Globally, Indigenous health workers are in a unique position to deliver culturally competent oral healthcare because they have a contextual understanding of the needs of the community.

**Methods:**

This scoping review aimed to identify the role of Indigenous health workers in promoting maternal oral health globally. A systematic search was undertaken of six electronic databases for relevant published literature and grey literature, and expanded to include non-dental health professionals and other Indigenous populations across the lifespan when limited studies were identified.

**Results:**

Twenty-two papers met the inclusion criteria, focussing on the role of Indigenous health workers in maternal oral healthcare, types of oral health training programs and screening tools to evaluate program effectiveness. There was a paucity of peer-reviewed evidence on the role of Indigenous health workers in promoting maternal oral health, with most studies focusing on other non-dental health professionals. Nevertheless, there were reports of Indigenous health workers supporting oral health in early childhood. Although some oral health screening tools and training programs were identified for non-dental health professionals during the antenatal period, no specific screening tool has been developed for use by Indigenous health workers.

**Conclusions:**

While the role of health workers from Indigenous communities in promoting maternal oral health is yet to be clearly defined, they have the potential to play a crucial role in ‘driving’ screening and education of maternal oral health especially when there is adequate organisational support, warranting further research.

## Background

Globally, Indigenous populations experience inequality in health status across the lifespan compared to their non-Indigenous counterparts [1]. While the factors which contribute to these inequities vary across continents, the inherent issues are remarkably similar. They are attributed to a combination of socioeconomic factors, colonisation, globalisation, migration, trans-generational loss of culture and disconnection from the land [2]. Although there have been some improvements in health outcomes in recent years, the gap in Australia remains significant [3].

In Australia, the Aboriginal and Torres Strait Islander people have poorer outcomes in maternal and infant health. A number of reports focusing on Indigenous people in Australia, New Zealand, Canada and the United States have shown that among pregnant women, the rates of gestational diabetes and the risk of developing Type 2 diabetes are higher compared to non-Indigenous women [4, 5]. Further, infants who are born to Australian Aboriginal and Torres Strait Islander mothers are also more likely to be born pre-term and of low birth weight [6] compared to Australians of other descents. In addition, Aboriginal and Torres Strait Islander children are twice as likely to develop early childhood caries (ECC) in their deciduous teeth and caries in their permanent dentition, compared to other children [7]. ECC is the most common chronic childhood disease worldwide and affects various aspects of the child’s functioning and quality of life [8]. Although typically overlooked, some evidence supports that maternal oral health may be associated with these health outcomes [9, 10].

During pregnancy, physiological factors such as hormonal variations, incidence of nausea and vomiting and dietary changes, increase the risk of pregnant women to dental problems such as dental decay and periodontal diseases [11, 12]. This is further exacerbated by sub-optimal diabetes control that has been associated with higher salivary glucose secretion, thus contributing to plaque accumulation [13, 14]. Poor maternal oral health has also been linked with increased adverse birth outcomes such as preterm birth, low infant birth weight and preeclampsia [15, 16]. Periodontal disease has also been shown to affect glycaemic control and contribute to diabetes complications [17]. Furthermore, maternal dental decay can also contribute to ECC as some babies can acquire their oral flora from carers, especially mothers, in addition to fomites such as feeding utensils which can facilitate bacteria transmission [18, 19].

Indigenous Australian pregnant women have a higher prevalence of dental pain relative to the general population and are more likely to avoid certain food due to these problems [20]. In light of the importance of maternal oral health, all pregnant women in Australia are encouraged to have an oral health check and education with their antenatal care provider, including treatment with a dental professional if required [21], similar to the United States, whereby women are advised to consult a dentist early during pregnancy and are provided oral health education, risk assessment and referral during antenatal care [22]. Regrettably, Indigenous mothers are more likely to attend their first antenatal visit later in the pregnancy, and these visits are less frequent than that of non-Indigenous pregnant women [23, 24]. The literature also reports a trend of underutilisation of preventive health services, including dental services [25, 26]. While financial cost is a main barrier to health service utilisation for this population [27], underlying social and historical factors continue to contribute to their distrust of healthcare professionals and the health system [28].

In view of the current challenges in accessing oral healthcare for Indigenous pregnant women worldwide, it is timely to explore alternative care providers to facilitate dental health-seeking behaviours. Indigenous Health Workers (IHWs) are well-placed to fill this gap because of their insight into the specific cultural needs and protocols of their communities, the importance of family networks and the impacts of colonisation on their health status and access to health services. IHWs in Australia originated from the Northern Territory during the 1950s where they were initially professionally employed as leprosy health workers, and later segued into their role as medical assistants. During the 1970s, they were recognised as important cultural brokers within their communities [29] as they were able to bridge the disconnect with non-Indigenous health professionals, and facilitate the process of accessing mainstream services by crossing the cultural barriers [29, 30]. This resulted in the development of the Aboriginal-controlled health services in Australia. In the non-dental health context, IHWs in Australia have been successful in leading culturally-appropriate health promotion and educational activities, in addition to being advocates for Indigenous communities [31–33]. Nevertheless the roles and functions of IHWs are often not well-defined as their roles are usually contingent on the local community’s needs, crossing the boundaries of clinicians, social support workers, cultural mentors and managers [29]. Despite the effectiveness in promoting primary healthcare among their Indigenous communities, a paucity in awareness by other healthcare workers, employers and organisations of their broad skillset may have contributed to the limited opportunity for training and career development [29]. Thus, the role of IHWs in promoting oral health for pregnant Indigenous women has received little attention.

### Aim

This review aimed to identify the potential role of IHWs in promoting oral health during the antenatal period. Specifically, this review sought to address the following research areas:

1. The evidence of the role of IHWs in promoting maternal oral health within Indigenous communities.
2. Training programs available to assist IHWs in oral health promotion during the antenatal period.
3. Available oral health screening tools that can be used by IHWs for women during the antenatal period.

### Terminology

In this review, the term *Indigenous health worker* was used to include health workers from Indigenous communities globally, and referred to persons who work within communities and act as the bridge between consumers and healthcare providers. They facilitate access to health services within communities and assume a role in health promotion and provision of culturally sensitive services [34, 35]. The term encompasses other terms like *health worker*, *community health worker*, *Aboriginal health worker* or *Aboriginal liaison officer.* In Australia, the terms *Aboriginal health worker* or *Aboriginal and Torres Strait Islander health worker* are used rather than *Indigenous health worker*. The term *non-dental health professionals,* refers to all health professionals other than dental professionals.

## Methods

### Study design

To source the available evidence regarding the role of IHWs in promoting oral health within Indigenous communities, training programs available to perform their role and functions, and the available oral health screening tools, we undertook a scoping review, using the framework described by Arksey and O’Malley [36]. This framework provided the structure to investigate the extent, range and nature of existing research, summarise the evidence, and identify any gaps within the literature. Unlike a systematic review, scoping reviews do not focus on the quality of the research which can significantly limit the type of studies included, enabling a broad range of literature, including reports, clinical guidelines, consensus statements, qualitative and quantitative studies [36]. Another advantage of a scoping review is the iterative process of going back-and-forth and redefining the study aims and search strategies based on the initial findings. This flexibility enables the researcher to gather a broad range of studies to inform the study topic, particularly suitable for topics with limited published evidence in the literature.

### Search strategy

A preliminary search was conducted with the assistance of a librarian for the following databases: CINAHL, Medline (Ovid), PubMed, ProQuest, Scopus and the Australian Public Affairs Information Service – Aboriginal and Torres Strait Islander Subset. We developed an individualised search strategy for each database according to their indexing terms. Boolean operators, truncations and Medical Subject Headings (MeSH) were also included to accommodate variations in spelling and terminology across countries. Reference lists of key articles were also searched for relevant literature. A search for grey literature through government and non-government organisations was also performed. Search strategies were derived from keywords which included: Aboriginal/Indigenous health worker, community health worker, maternal infant care worker, non-dental/oral health professional, oral health, oral hygiene, dental care, training/education program, oral/dental assessment, pregnant, antenatal, perinatal and assessment/ screening tool.

### Inclusion and exclusion criteria

For this review, we selected all articles published up to July 2017 relating to at least one of the research areas. Except for discussion papers, reviews and study protocols, research papers of all other study designs were included. No restrictions were placed on the quality or location of the study, however, studies which focussed on dental professionals promoting oral health among Indigenous pregnant women, and those not published in the English language were excluded.

### Study selection and data extraction

**Fig. 1:**
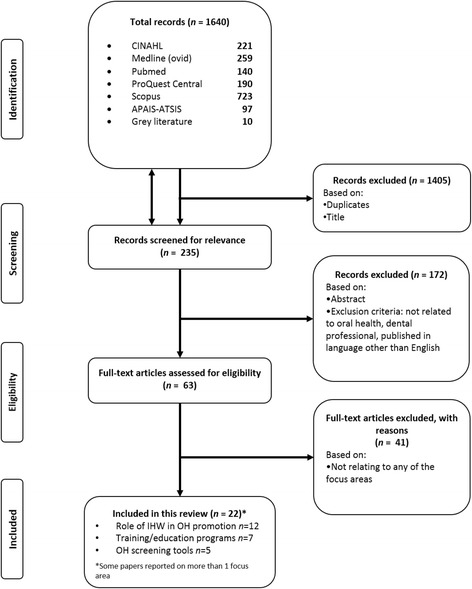
PRISMA flow chart illustrating the search strategy

The data were extracted from the selected studies and categorised under the following headings: author/study location; article type; aims; study design; intervention/program/screening tool; conclusion and focus area. These were subsequently categorised into the three focus areas corresponding to the research question. The first focus area investigated studies where IHWs undertook a role in promoting oral health among pregnant women. The second and third foci explored studies that described any oral health education/training programs and screening tools developed for IHWs in the antenatal setting. If the initial search across all three focus areas revealed limited or no studies, the search was expanded to IHWs promoting oral health in other settings and all non-dental health professionals involved in maternal oral healthcare (Fig. 1).

## Results

Database searches yielded 1640 records. After 1405 duplicates and records screened by titles were excluded, 235 abstracts were screened for relevance; identifying 63 pertinent records. The full texts of the 63 papers were reviewed using inclusion and exclusion criteria. Three government-published documents were also included. Twenty-two articles were identified in this scoping review in relation to the potential role of IHWs in promoting oral health, training programs and potential oral health screening tools that can be used by IHWs for women during the antenatal period (Fig. 1). Studies were based in the United States (*n* = 7), Canada (*n* = 2), Australia (*n* = 11), Turkey (*n* = 1), and India (*n* = 1). These papers were clustered into three broad focus areas: i) role of IHWs in promoting maternal oral health (*n* = 12); ii) antenatal oral health training programs for IHWs (*n* = 7); and iii) potential antenatal oral health screening tools suitable for IHWs (*n* = 5) (Table 1).

**Table 1:** Articles that relate to the potential role of IHWs in promoting maternal oral health

Author/Study Location	Article Type	Aims	Study Design	Intervention/Program/Screening Tool	Conclusion	Focus Area
Adams et al., 2017 [44] United States	Peer-reviewed journal article	Evaluate effectiveness of a low-cost educational intervention program in clinically improving oral health (OH)	Non-randomised controlled trial	Intervention: • CenteringPregnancy Oral Health Promotion program (delivered by midwives) • 3-h training course for midwives • Post-training practice session with feedback.	• Significant improvement in clinical OH outcomes as indicated by mean plaque index (*p* < 0.001), sites bleeding on probing (*p* = 0.01) & pocket depths (*p* < 0.001)	• Potential role of IHW • Training program
AIHW, 2011 [39] Australia	Government report	Reports on dental data collected from the Closing the Gap Child Oral Health Prevention and Promotion program in the Northern Territory (2009–2010)	N/A	Intervention: • Trained registered nurses and AHWs to apply fluoride varnish to at-risk children every 6 months • Child health checks, conducted by nurses/medical staff, for referrals and improving access to dental services	• Children who received a health check by nurses or medical staff, who also performed a Lift the Lip assessment, could be referred to the dental service for free • 37% of children (< 16 yrs) in the communities received a dental service	• Potential role of IHW
Braun et al., 2016 [40] United States	Peer-reviewed journal article	Assess effectiveness of an OH promotion program in reducing caries increment in Navajo children	Cluster-randomised trial	Intervention: • Community tribal members trained as community OH specialists • Delivered OH intervention in classrooms and to families	• Caries rate (including decayed, missing and filled surfaces): Nil significant change in children • Caregiver OH knowledge & behaviour: Rapid improvement after 1 year; no difference after 3 years between groups	• Potential role of IHW
Cibulka et al., 2011 [47] United States	Peer-reviewed journal article	To evaluate the effectiveness of advanced practice nurse model of care to improve the OH among low-income pregnant women	Randomised controlled trial	Intervention: • Video with discussion on OH conducted by nurses • Distributed an oral hygiene kit (toothbrush, fluoridated toothpaste and dental floss).	• No significant change in OH knowledge/perceptions of pregnant women between baseline and follow-up at 36 weeks • Improved OH practices (e.g. frequency of brushing and flossing teeth) and attendance for dental check-up during pregnancy • Reduced OH problems in 3rd trimester	• Potential role of IHW
Dental Health Services Victoria, 2017 [41] Australia	Government brochure	To provide culturally-appropriate information about available dental services for Aboriginal and Torres Strait Islander peoples	N/A	Intervention: • Free dental services for any Aboriginal or Torres Strait Islander person at the Royal Dental Hospital of Melbourne	• Aboriginal Liaison Officer can assist with streamlining communication	• Potential role of IHW
Deshpande et al., 2015 [54] India	Peer-reviewed journal article	Assess the impact of the perinatal OH care education program on the knowledge, attitude & practice behaviour amongst gynaecologists	Cross-sectional	Program: • Flip chart and OH resource brochures provided to 46 gynaecologists • Assessed after 1 month	• Significant improvement in OH knowledge (*p* < 0.001), & practice behaviour (*p* < 0.001) • No significant change (*p* = 0.49) in attitude of respondents	• Training program
George et al., 2016 [52] Australia	Peer-reviewed journal article	Evaluate the effectiveness of the Midwifery Initiated OH (MIOH) program in improving the OH knowledge of midwives & assess their confidence to promote maternal OH post training	Pre-post test	Program: • Antenatal OH education and referral • 3 self-paced online modules over 3 months Delivered to 50 midwives	• Significantly improved midwives’ knowledge (*p* < 0.001) • At program completion 82% of respondents were confident in introducing the topic of OH in their antenatal session, 77.6% were confident with dental service referrals, and 46% were confident to undertake a visual mouth check	• Training program
George et al., 2016 [56] Australia	Peer-reviewed journal article	Undertake sensitivity and specificity assessment of the maternal OH screening tool using two comparison approaches- the Oral Health Impact Profile and a clinical oral assessment by trained study dentists	Diagnostic test	Screening tool: 2-Item Maternal Oral Health Screening Tool administered by midwives 1. OH status 2. OH risk factors	• High sensitivity against the gold standards measured (93.3%, 88.2–97.9% CI) • Low specificity (20.5%, 13.2–27.8% CI) • Tool reliable to screen and refer women with OH problems to the dentist	• Screening tool
Hunter et al., 2011 [57] United States	Peer-reviewed journal article	Describe the OH status and OH practices of low-pregnant women in San Diego, California, and determine the needs for OH care education in this population	Descriptive correlational	Screening tool: 12-Item Oral Health Assessment Questionnaire administered by bilingual (English/Spanish-speaking) nurse-midwives 1. OH status 2. OH risk factors 3. Dietary risk factors	• Poor OH (prevalence of tooth decay was 45.9% and gingivitis was 36.7%) • Sample less likely to access dental services although had some good daily OH practices • Highlighted need for additional OH education	• Screening tool
Johnson et al., 2013 [45] Australia	Conference abstract	To evaluate the effectiveness of the midwifery-initiated oral health dental service (MIOH-DS) program in improving the uptake of dental services, quality of life and OH knowledge among pregnant women	Randomised controlled trial Three groups: 1) No intervention; 2) Midwifery Intervention (MIOH); and 3) Midwifery and dental intervention (MIOH-DS)	Intervention: • OH education, assessment and priority dental referrals during early pregnancy • Delivered by midwives	• 50% improvement in dental service uptake for participants who received the midwifery-initiated oral health dental service (MIOH-DS) intervention program • No significant difference between group receiving MIOH intervention and control group • Quality of life significantly improved in both intervention groups • OH knowledge significantly improved for all 3 groups	• Potential role of IHW
Lawrence et al., 2017 [48] Canada	Peer-reviewed journal article	Assess effectiveness of the Sioux Lookout Zone prenatal program on primary caregivers’ dental preventive beliefs, behaviours and feeding habits of infants and toddlers	Longitudinal and cross-sectional approaches	Intervention: • One-on-one, culturally-appropriate, nutrition and OH preventive education • Woman-and-child nutrition educators visited caregivers in their homes	• High program coverage (> 70% community received intervention) had significantly improved dental knowledge (*p* < 0.05) and practices (e.g. toothbrushing frequency) • Over 90% of children were found to have early childhood caries despite changes in knowledge, beliefs and practices	• Potential role of IHW
Mathu-Muju et al., 2016 [38] Canada	Peer-reviewed journal article	To increase access to preventive dental services for First Nations and Inuit children living on federal reserves & in remote communities of Canada	Cross-sectional	Intervention: • Children’s Oral Health Initiative (COHI) provided preventive dental care with culturally-appropriate OH messages • Dental therapists and hygienists collaborated with an Indigenous COHI aide	• Increased access of preventive dental services from 2006 to 2014 • Community capacity building with the employment of COHI aide was successful in improving access to preventive dental care. Approximately 50% of children living in the reserves participated in the OH initiative.	• Potential role of IHW
McGuire et al., 1998 [49] Australia	Peer-reviewed journal article	To describe the OH workshop targeting AHW (Aboriginal health worker) trainees in a remote community	Descriptive	Program: • OH program (2 day workshop) with 23 AHW trainees • Focussed on dental conditions, OH knowledge & education, prevention & treatments (fissure sealants, fluoride, oral hygiene products)	• Described as interactive, practical & engaging • Reported to be valuable for those in remote areas	• Training program
New York State Department of Health, 2006 [58] United States	Clinical practice guidelines	Develop clinical practice guidelines for health care professionals relating to OH care for pregnant women and young children	N/A	Screening tool: 2-Item questionnaire for initial prenatal screening administered by antenatal care provider 1. OH status 2. OH risk factors	• Discussed and outlined role of antenatal providers to integrate OH into maternal health	• Screening tool
Öcek et al., 2003 [53] Turkey	Peer-reviewed journal article	Evaluate the effectiveness of a dental health program for midwives working in primary health care services	Mixed-methods	Program: • Interactive OH educational program focussed on infants • Content based on pre-test assessment • Program delivered to 164 midwives	• Improvement in OH knowledge among midwives (significance unreported) • Program perceived to be relevant to practice	• Training program
Oral Health Care During Pregnancy Expert Workgroup, 2012 [22] United States	National consensus statement	Developed to assist health professionals, program administrators and staff, policymakers, advocates, and other stakeholders respond to the need for improvements in the provision of OH services to women during pregnancy	N/A	Screening tool: 4-Item oral health questionnaire administered by prenatal healthcare professionals 1. OH status 2. OH risk factors 3. Visual inspection	• Questionnaire developed from a national expert panel coordinated by the National Maternal and Child Oral Health Resource Center	• Screening tool
Pacza et al., 2001 [51] Australia	Peer-reviewed journal article	Institute a culturally appropriate preventative OH program at a community level	Descriptive survey	Program: • Pilot OH training program for 27 AHWs in a rural and remote community • 3 modules over 96 h (36 h in the classroom; 60 h on-the-job)	• Material was relevant & enjoyable • Difficulty was moderate • Students perceived they had a good understanding of module objectives and relevant to their needs.	• Training program
Parker et al., 2005 [42] Australia	Peer-reviewed journal article	Describe the development, implementation and evaluation of the first stage of the OH Program (dental clinic/service) for the Indigenous community serviced by Pika Wiya Health Service	Descriptive	Intervention: • Rural OH service designed to meet cultural needs of the Indigenous population • Recruited an AHW to coordinate for administrative duties and establishing the program	• High service demand in 1 year (229 individuals, 1582 treatments) • AHW involved in program development, health promotion & recruitment	• Potential role of IHW
Slade et al., 2011 [43] Australia	Peer-reviewed journal article	To evaluate effectiveness of trained primary healthcare workers in preventing dental caries in preschool children living in remote Aboriginal communities in Northern Territory	Cluster-randomised, concurrent controlled trial	Intervention: • Nurses and/or AHWs applied fluoride varnish on children, advised parents on caries prevention, promoted traditional health, demonstrated OH practices • Engaged members of community during events.	• Intervention consisting of fluoride varnish & OH promotion reduced caries by 24–36%	• Potential role of IHW
Smith et el., 2016 [50] Australia	Peer-reviewed journal article	To evaluate the OH training program targeting AHWs in cultural appropriateness, course content and respondents’ perception of competence to offer OH advice	Qualitative	Program: • Smiles not Tears OH programme (1 day with presentation, role-play & group discussion) • Delivered to 61 AHWs educating about OH for young children	• Increased AHWs’ confidence to offer dental advice to target population • Course received positive feedback in content and perceived to be culturally appropriate	• Training program
South Australia Dental Service, 2015 [37] Australia	Business Plan	Improve oral health outcomes for eligible Aboriginal and Torres Strait Islander people in South Australia by increasing the number who access mainstream dental services	N/A	Intervention: • Aboriginal Oral Health Program trained Health Workers to assess, refer adults, children, and pregnant women to dental services • Developed a streamline referral pathway for pregnant women	• Outlined seven key performance indicator objectives and corresponding actions/strategies	• Potential role of IHW
Stevens et al., 2007 [46] United States	Peer-reviewed journal article	Describe strategies used by one adolescent pregnancy program to implement New York State Department of Health (NYSDOH) OH guidelines	Descriptive	Intervention: • Nurse midwives and a nurse practitioner educated, assessed and referred patients to dental services Screening Tool: Two-item questionnaire (NYSDOH 2006)	• Dental risks screening, referrals, education & regular dental care were vital to program • Nurses were “driving force” for OH promotion; they led the screening, assessment & education	• Potential role of IHW • Screening tool

### Focus area 1: Role of IHWs in promoting maternal oral health

Within this focus area, three categories of literature were identified that could inform the potential role of IHWs in promoting maternal oral health: i) IHWs who have promoted maternal oral health; ii) IHWs who have supported oral health across the lifespan; and iii) non-dental health professional who have promoted maternal oral health.

#### IHWs in promoting maternal oral health

Two articles identified the contribution of IHWs in promoting maternal oral health in an Australian [37] and Canadian context [38]. The Children’s Oral Health Initiative (COHI) in Canada aimed to reduce rates of ECC among Indigenous communities through oral health promotion, screening and preventive dental treatment; IHWs were involved in scheduling dental appointments, applying the fluoride varnish and giving one-on-one oral health education to children, expectant mothers and parents in home-based visits. The Aboriginal Oral Health Program in South Australia [37] developed and streamlined, state referral pathway that involved health workers assessing and referring Australian Aboriginal and Torres Strait Islander pregnant women to dental services. Details of the assessment or their training was not described.

#### IHWs in supporting oral health across the lifespan

Five studies reported the role of IHWs in providing oral healthcare for Indigenous populations across the lifespan, ranging from early childhood (18 months to 5 years) to elderly [39–43]. Parker et al. [42] evaluated the implementation of a free Indigenous dental clinic in a remote Australian community. Although the roles of IHWs were not clearly defined, they were involved in program development and recruitment. This initiative was well received with a high demand over the year it was funded for implementation. The Dental Health Services in Victoria [41] published a brochure informing clients that free, public dental services were available for Indigenous Australians at a major dental hospital. Aboriginal liaison officers were featured as contact personnel to assist in brokering and accessing services. The Australian Institute of Health and Welfare [39] reported that IHWs applied fluoride varnish on children within communities in the Northern Territory and assisted in contacting families as part of the national health campaign, Close the Gap. Among the communities targeted, 37% of children received a dental service. One Australian [43] and one cluster-randomised trial in the United States [40] also trained IHWs or community tribal members to deliver oral health education and fluoride varnish applications at preschools and within Indigenous communities, respectively. Slade et al. [43] trained Indigenous Australian health workers to assess and refer preschool children with dental decay, and reported reduced incidence of dental caries. No significant change to the children’s oral health status as measured by the number of decayed, missing or filled surfaces was reported in the study conducted in the United States [40].

#### Non-dental health professionals in supporting maternal oral health

Five studies were identified that described the role of non-dental health professionals in promoting maternal oral health, including midwives [44, 45], nurses practitioners [46], advance practice nurses [47] and maternal and child nutrition educators [48]. Two studies involving midwives discussed oral health topics with pregnant women, including demonstrating proper oral hygiene techniques, performing oral health screening and providing priority referrals to dental services. In these studies significant improvements were observed in clinical oral health outcomes [44], dental service uptake, self-reported quality of oral health and oral health knowledge of pregnant women. The oral health knowledge and confidence of midwives to promote oral health also showed marked improvement [45]. The New York State Department of Health oral health guidelines, implemented by Stevens et al. [46], described the use of midwives and nurse practitioners to provide oral health screening, education and referrals to dental services; nonetheless, effectiveness of the program was not reported. Another study employed advance practice nurses to improve oral health of low-income pregnant women in the United States. Although this program did not significantly improve women’s knowledge, significant improvements in oral health practices and dental service uptake were reported, as well as a reduction in the rates of oral health problems in late pregnancy [47]. Oral health practices were also improved in Canada where maternal and child nutrition educators provided one-on-one dental preventive education to the whole community, including pregnant women [48]. Despite improvements in practice, the prevalence of ECC remained high.

### Focus area 2: Antenatal oral health training programs for IHWs

Within focus area 2, three categories of literature were identified that could inform the training programs for IHWs: i) IHWs training programs to promote maternal oral health; ii) IHWs training programs to support oral health across the lifespan; and iii) training programs for non-dental health professionals in promoting maternal oral health.

#### IHWs training programs to promote maternal oral health

Mathu-Muju et al. [38] briefly described a Canadian government-initiated training program (COHI) for IHWs to enable them to provide oral health education primarily for children, parents, caregivers, but also for expectant mothers. IHWs were trained in five areas not specifically defined in the study.

#### IHWs training programs to support oral health across the lifespan

This scoping review identified four Australian-based studies reporting different oral health training programs for IHWs to support Indigenous populations across the lifespan [43, 49, 50]. Two of the programs were introduced specifically to address the needs of communities in rural and remote areas [51]. Pacza et al. [51] in consultation with stakeholders from the local Indigenous medical service developed a three-module training program delivered in a classroom in addition to 60 h of on-the-job training. The modules covered an introduction to dental health and knowledge, applied oral health, and application to their local community. Another two studies delivered training through a one or 2 day workshop including education on oral health, oral hygiene and preventive treatments, emphasising the IHWs’ utilisation of role-play, which received positive feedback [49, 50]. Smith et al. [50] also trained IHWs to use an early childhood oral health screening tool. Finally, one study [43] involved training IHWs to reduce ECC incidence through oral disease recognition, referral to dental services, oral health promotion, fluoride varnish application, provision of chart books and DVD instructions; however, mode of delivery and evaluation were not clearly described.

#### Training programs for non-dental health professionals in promoting maternal oral health

The search yielded four articles reporting training programs in promoting maternal oral health to non-dental health professionals including midwives [44, 52, 53] and gynaecologists [54]. Two of these studies delivered face-to-face oral health education and promotion programs [44, 53] while the other two employed an online self-paced module [52] and written material [54] as the delivery mode of training activities. Öcek et al.’s program in Turkey [53] increased oral health knowledge of midwives through a face-to-face program involving oral hygiene procedures, techniques, and eruption chronology of deciduous teeth in young children. Adams et al. [44] did not include an evaluation report, however, their face-to-face training in the United States for midwives included education on the importance of maternal oral health and dental care during pregnancy and instructions on oral health promoting practices including flossing and tooth-brushing, and was developed to be integrated into pre-existing antenatal sessions. George et al. [52] delivered and evaluated an online self-paced three-module program designed with theoretical and practical oral health content for midwives in Australia. The modules enhanced antenatal oral health knowledge, and also trained midwives to use an antenatal oral health screening tool which assisted their referrals to dental services. Deshpande et al. [54] also demonstrated an improvement in oral health knowledge with gynaecologists in India by means of a flip-chart and oral health brochures.

### Focus area 3: Antenatal oral health screening tools developed for IHWs

This scoping review could not identify any oral health screening tools developed specifically for IHWs in any setting. Nevertheless, there is one Australian report of IHWs being able to utilise an existing early childhood oral health screening tool, ubiquitously known as ‘Lift the Lip’, to identify ECC through a combination of parental concern and visual inspection [50]. IHWs could refer appropriately during a role-play demonstration, and reported the tool to be culturally acceptable. This resource has also been adapted for Australian Aboriginal and Torres Strait Islander families, and renamed as ‘See My Smile’ [55].

Five papers were identified that described an antenatal oral health screening tool for non-dental health professionals [22, 46, 56–58]. A descriptive study in the United States provided the 12-item oral health questionnaire which was utilised by bilingual nurse midwives [57]. The questionnaire was assessed for cultural relevancy and currency by six pregnant women prior to its administration. The items identified the patient’s health-seeking behaviours including dental service uptake, oral health status such as dental or gum pain, and risk factors such as vomiting frequency. Furthermore, a national consensus statement in the United States proposed a four item oral health questionnaire to be used by prenatal healthcare professionals [22]. This tool similarly aimed to identify oral health status, oral health risk factors and health-seeking behaviours. Another shorter screening tool developed for antenatal care providers, consisting of two items focusing on identifying oral health problems and visits to the dentist in the previous 6 months, was published in the New York State Department of Health [58] clinical guidelines. Stevens et al. [46] demonstrated that nurse midwives and nurse practitioners were able to utilise this screening tool, but no formal evaluation was conducted. An almost identical two-item screening tool assessing whether the pregnant woman had any dental problems, and whether they have visited a dentist in the last 12 months was developed and evaluated for midwives in Australia [56]. This tool was validated against two gold-standard assessments: a clinical evaluation by trained dentists and a subjective oral health assessment. It demonstrated high sensitivity in detecting women who required a dental visit.

## Discussion

A review of the literature indicated that some IHWs have had roles in improving oral health outcomes of pregnant women, particularly in South Australia. However, there are currently no ‘proof of concept’ interventions, training programs or screening tools that can provide IHWs with the knowledge and skills to promote maternal oral health and refer pregnant women appropriately to dental services. While one study by Mathu-Muju et al. [38] trained IHWs to promote oral health among pregnant women, it focussed on reducing rates of ECC in children.

Although there is some evidence of maternal oral health training programs and screening tools available for non-dental health professionals, certain issues require consideration prior to their use by IHWs. An important factor to consider is whether these interventions are culturally appropriate. Kreuter and colleagues [59] identified cultural appropriateness to be an essential element in interventions for Indigenous populations to reflect their unique health values, practices and behaviours. Another factor is that these programs were designed for individuals with a specific skill set to undertake oral assessments and provide oral health education. Consequently, there may be an incongruity between the educational background of IHWs and the clinical care requirements of these programs. For instance, some of the programs and tools were designed for midwives, nurses or other health professionals who typically complete tertiary level education [60]. However, in Australia, IHWs may not necessarily have the same degree of educational qualification as many complete vocational education only [61].

Despite this, a university-level education may not be a necessary pre-requisite to raise oral health awareness or to provide clinical care. In Australia, the United States and Canada, IHWs encouraged oral health within communities and played integral roles in applying fluoride varnish [38, 40, 43, 49]. Various other studies have similarly demonstrated the key role of an IHW in breaking down barriers and bridging the gap in other healthcare settings. In the Australian state, Victoria, an Aboriginal liaison officer was the key contact through whom communities could choose to engage with the service [41]. They have also acted as a foundational support and conduit in the provision of care in mental health and cardiovascular settings where they developed mental health knowledge and assessment skills; improved Indigenous people’s transition to mental health services; and more effectively delivered cardiovascular health education and care in hospitals with the expertise of other health professionals [62–64]. These studies highlight the capacity of IHWs to educate, assess and assist pregnant women to navigate through the healthcare system.

The literature also emphasises their role in brokering between communities and other healthcare professionals, including services with GPs, midwives, nurses and dentists. Walker and colleagues [65] explored the perspectives of health personnel, excluding IHWs, on the oral health role of IHWs. Their findings demonstrated that dental and non-dental health personnel perceived IHWs to be in an important position to effectively promote oral health within their communities. Another study conducted with IHWs suggested that appropriate oral health training should be given by a team of IHWs and dentists to relay the relevant information to IHWs [66]. They also highlighted that training needed to be adopted by the team and that there should be adequate management support.

Models of care that enable effective partnerships between Indigenous women and IHWs, and that focus on continuity of care ensure that cultural needs are respected during this transition into parenthood. IHWs are integral to these partnerships as they share the same “language” as their community which facilitates effective communication [67]. This common language encourages compliance to interventions and uptake of available health services, including dental services, within the healthcare system [29, 68]. Having the same language, fosters a relationship where patients feel a sense of empowerment and feel included in decision-making during the perinatal period, which further enhances self-efficacy [69]. Conway and colleagues [70] attributed the success of their program, which aimed to provide holistic, patient-centred chronic care management to Indigenous Australians, to its focus on the patient’s story; providing patients the opportunity to make decisions about their own health.

Another important aspect in developing models of care in this area is to ensure the Aboriginal community is ready to receive oral health information from AHWs. One way of addressing this is to use motivational interviewing (MI). MI is a client-centred counselling intervention designed to elicit intrinsic behavioural change [71]. MI techniques have demonstrated significant effectiveness in improving health outcomes including blood pressure, cholesterol and body mass index [72].

Lastly, for effective transitional care and effective partnership models of care, there needs to be adequate support from the healthcare system. This may require organisational changes and sufficient resources and personnel to enact change [73, 74]. In Australia, free dental care generally is available through the public healthcare system for adults with low income. Although eligibility may not be conditional to Aboriginal and Torres Strait Islander status depending on the state, these individuals receive priority access [75]. Despite priority access, a high proportion of Aboriginal and Torres Strait Islander Australians do not regularly attend a dental service [76]. Campbell and colleagues [77] identified several barriers which can affect their access to health services which include long waiting times, distance, lack of transportation and previous experiences of racism. The lack of recruitment of Aboriginal and Torres Strait Islander staff in the healthcare system is another cited problem. It is recognised that to consolidate the Aboriginal health workforce requires specific support for the challenges they are likely to face including racism, community responsibilities, family issues, sense of isolation and stress and poor education [78]. To improve access, some Aboriginal Health Services, which provide free healthcare to their communities, are funded to provide dental care [75]. However, as not all Aboriginal Health Services receive funding for dental care, it is important to explore strategies to increase the number of Aboriginal and Torres Strait Islander dental staff and link their community to public dental services. The literature has shown great potential for capacity building IHWs to provide culturally-appropriate oral healthcare and referral to dental services within the healthcare system for pregnant women with adequate organisational support and resources.

There were a number of limitations in this review that need to be acknowledged. The strength of the evidence of the intervention studies which included IHWs were poor based on their study design quality [79] while the grey literature included may not necessarily be critically peer-reviewed. Post-hoc analyses were frequently utilised in studies and few conducted significance testing. A possible explanation for this might be associated with the challenges of developing culturally-appropriate training programs and tools since considerable time may be necessary to negotiate and build partnerships with Indigenous communities [32]. There is also the ethical challenge of conducting rigorous research while ensuring that healthcare is not compromised for this vulnerable population. It was also difficult to define the role of IHWs from country to country which therefore limited comparisons. Further, there was a paucity of evidence comparing Indigenous populations with other disadvantaged groups, which may be a more useful comparison in identifying sociocultural barriers in future studies.

### Recommendations

Capacity building IHWs in maternal oral health requires both change to policy and practice. A structured framework incorporating organisational change and the allocation of sufficient resources may need to be implemented to strengthen oral health referral pathways from antenatal settings. Oral health interventions and protocols should be developed in partnership with IHWs to ensure training programs and tools are validated, pragmatic, culturally appropriate, easy to use, and accommodate continuity of care. Further, to address the needs of IHWs in both urban and remote areas, training programs may need to be designed to train-the-trainer or to adopt telehealth models.

## Conclusions

IHWs over the globe have had some role in promoting oral health in the antenatal setting. Further work is necessary, however, to develop validated antenatal oral health training programs and screening assessment tools that respect Indigenous cultural values. More importantly, models of care that demonstrate and foster mutual partnerships that assist Indigenous peoples in accessing dental care require further research for implementation across the population.

## Abbreviations

AHW: Aboriginal health worker
COHI: Children’s Oral Health Initiative
ECC: Early childhood caries
IHW: Indigenous health worker
MeSH: Medical Subject Headings
MIOH: Midwifery initiated oral health
MIOH-DS: Midwifery initiated oral health dental service
OH: Oral health
